# Bottom-Up Fabrication of Protein Nanowires via Controlled Self-Assembly of Recombinant *Geobacter* Pilins

**DOI:** 10.1128/mBio.02721-19

**Published:** 2019-12-10

**Authors:** K. M. Cosert, Angelines Castro-Forero, Rebecca J. Steidl, Robert M. Worden, G. Reguera

**Affiliations:** a Department of Microbiology and Molecular Genetics, Michigan State University, East Lansing, Michigan, USA; b Department of Chemical Engineering, Michigan State University, East Lansing, Michigan, USA; University of Washington

**Keywords:** type IV pili, microbial nanowires, nanotubes, synthetic biology, electromicrobiology

## Abstract

Metal-reducing bacteria in the genus *Geobacter* use a complex protein apparatus to guide the self-assembly of a divergent type IVa pilin peptide and synthesize conductive pilus appendages that show promise for the sustainable manufacturing of protein nanowires. The preferential helical conformation of the *Geobacter* pilin, its high hydrophobicity, and precise distribution of charged and aromatic amino acids are critical for biological self-assembly and conductivity. We applied this knowledge to synthesize via recombinant methods truncated pilin peptides for the bottom-up fabrication of protein nanowires and identified rate-limiting steps of pilin nucleation and fiber elongation that control assembly efficiency and nanowire length, respectively. The synthetic fibers retained the biochemical and electronic properties of the native pili even under chemical fixation, a critical consideration for integration of the nanowires into electronic devices. The implications of these results for the design and mass production of customized protein nanowires for diverse applications are discussed.

## Abstract (precis)

The discovery in 2005 of conductive protein appendages (pili) in the metal-reducing bacterium Geobacter sulfurreducens challenged our understanding of biological electron transfer and pioneered studies in electromicrobiology that revealed the electronic basis of many microbial metabolisms and interactions. The protein nature of the pili afforded opportunities for engineering novel conductive peptides for the synthesis of nanowires via cost-effective and scalable manufacturing approaches. However, methods did not exist for efficient production, purification, and *in vitro* assembly of pilins into nanowires. Here we describe platforms for high-yield recombinant synthesis of *Geobacter* pilin derivatives and their assembly as protein nanowires with biochemical and electronic properties rivaling those of the native pili. The bottom-up fabrication of protein nanowires exclusively from pilin building blocks confirms unequivocally the charge transport capacity of the peptide assembly and establishes the intellectual foundation needed to manufacture pilin-based nanowires in bioelectronics and other applications.

## INTRODUCTION

Bacteria in the genus *Geobacter* assemble conductive protein appendages of the type IVa pilus class that protrude from the outer membrane to discharge respiratory electrons onto extracellular electron acceptors such as ferric iron (Fe[III]) oxides (1) and the uranyl cation (2). The *Geobacter* pili are an assembly of primarily one peptide, the PilA pilin (2), with the distinctive α-helix domain and amino acid sequence motifs of type IVa structural pilins (1, 3, 4). However, the *Geobacter* pilins are shorter than canonical type IVa pilins and form an independent line of descent from pilins of other members of the order *Desulfurococcales* (1). Consistent with their distinct phylogenetic placement, *Geobacter* pilins have a divergent structure and amino acid composition that allows them to assemble as a conductive fiber (4, 5). Nevertheless, pilin assembly follows the conserved steps of other bacterial type IV pili that recognize, process, and assemble the peptide into a pilus fiber (reviewed in reference 6). The model representative Geobacter sulfurreducens synthesizes two pilin precursors (prepilins) with a long or short signal peptide whose interactions permit optimal pilin export to the inner membrane and coregulation of cytochrome secretion (7). The two prepilin isoforms carry the conserved recognition sequences needed for removal of the leader peptide and *N-*methylation of the mature peptide by a conserved PilD prepilin peptidase (7). A canonical type IV pilus apparatus then assembles the pilins vertically on the inner membrane and through an outer membrane PilQ porin secretin, exposing the base of the pilus to the abundant periplasmic cytochromes to permit the discharge of respiratory electrons onto the conductive fibers (6).

Charge transport from the pili to the extracellular electron acceptors does not appear to be rate-limiting (8). A 1-μm-long pilus fiber, for example, can transport ∼1 billion electrons per second at biologically relevant voltages of 100 mV—2 orders of magnitude faster than the cellular respiration rate measured in iron oxide cultures of G. sulfurreducens (8). In addition, each cell assembles numerous pili on one side of the cell (1), providing many electronic conduits for the extracellular discharge of respiratory electrons. The conductive pili are also dynamic and protrude and retract to recycle the pilins and allow for multiple cycles of electron discharges (9). Antagonistic cycles of pilus protrusion and retraction are also important to remove minerals that remain bound to the fibers after their reduction (9). The reduction of iron oxides, for example, solubilizes part of the Fe(III) as Fe(II) but also generates magnetite, a magnetic mineral of mixed Fe(III)/Fe(II) valence that remains attached to the pilus fibers. Similarly, the pili reduce the uranyl cation to a mononuclear uranium mineral phase at their surface (2). To enable new rounds of respiration, cells use a conserved PilT ATPase (PilT4) to depolymerize the pilins at the base of the pilus and release the reduced minerals (9). Furthermore, depolymerization stores the pilin peptides in the inner membrane, making them readily available for a new round of polymerization in a reaction energized by a conserved PilB ATPase (9, 10).

**FIG 1:**
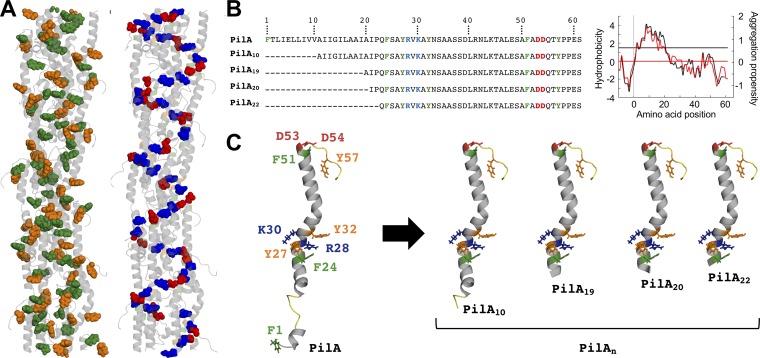
Structures and amino acid sequences of G. sulfurreducens pilus fiber (A) and pilins (B and C). (A) Molecular structure of a PilA pilus fiber optimized via molecular dynamics (MD) showing aromatic residues (phenylalanines in green, tyrosines in orange) and charged amino acids involved in salt bridges (acidic amino acids in red, basic amino acids in blue). (B) Positions of the aromatic and charged residues in the amino acid sequence of the PilA monomer and the N-terminal (N-t) truncated PilA_n_ derivatives. The graph on the right shows Kyte-Doolittle (black) and AGGRESCAN (red) plots of the PilA prepilin (pilin with signal peptide) and cutoff values (horizontal lines) of hydrophobicity and aggregation potential scores. (C) MD-optimized model of the mature PilA pilin and truncated (PilA_n_) derivatives highlighting aromatic and charged amino acids shown in panel A.

Studies of G. sulfurreducens have helped define structural features and amino acids of the *Geobacter* pilins that are critical for fiber formation and conductivity (reviewed in reference 6). The reduced size of most *Geobacter* pilins (61 amino acids in G. sulfurreducens compared to 142 to 175 in other bacterial pilins) results from the replacement of the carboxy-terminal (C-t) globular head of canonical type IVa pilins with a short, flexible random-coiled segment (3, 4). Consequently, *Geobacter* pilins lack the distinctive αβ-loop, antiparallel β-sheet domain, and D-region (with flanking cysteines) of canonical pilins, which confer on other pili their distinctive surface properties (11). Instead, the *Geobacter* pili expose on their surface a short and flexible C-t segment with amino acid ligands required for metal binding and reduction (5). The *Geobacter* pilins conserve, however, the amino-terminal (N-t) α-helix (α1 domain) that is essential for hydrophobic interactions leading to the formation of the pilus fiber core (5). Salt bridges between positively and negatively charged amino acids from neighboring α1-domains (D53-K30 and D54-R28) maintain the pilins tightly bound together and align them optimally to cluster aromatic amino acids at distances optimal for charge transport (5) (Fig. 1A). Molecular dynamics (MD) simulations of the pilus fiber predict some of the aromatic side chains of neighboring pilins to cluster within 3- to 5-Å distances, but these aromatic “contacts” never form at the same time, as in a metallic wire (5). Furthermore, the geometry of the aromatic contacts is displaced, that is, the aromatic rings are not aligned in sandwich type configurations that could permit π–π stacking and metal-like conductivity (5). Yet the interaromatic distances and geometries are optimal for the transport of charges via hopping. Consistent with this, charge mobility along the pilus fibers is too low to support a band conduction mechanism as in metals, and the pilus conductivity is thermally activated, a hallmark of charge hopping (8).

Harnessing the unique properties of *Geobacter* pili in biotechnology will ultimately require protocols for their production and functionalization at the yields and costs that are needed to satisfy market demands (6). Direct purification of the conductive pili from native cells is achievable yet requires many steps to separate the fibers from other cellular components and purify them free of impurities and contaminants (2, 8). Importantly, cultivation of piliated cells under anaerobic conditions and fiber isolation steps are not easily scalable (2, 8). Recombinant expression in fast-growing recombinant hosts such as Escherichia coli could alleviate these constraints, yet the toxic effects caused by the aggregative nature of the nanowire pilin, even when expressed with solubility tags, limit production (12). One way to overcome these challenges is to design recombinant systems that express pilins carrying N-t truncations that reduce the peptide’s hydrophobicity to levels that enable its expression as fusion proteins with solubility modules (12). In a previous study, we demonstrated the suitability of this approach for high-yield recombinant expression of a thiolated G. sulfurreducens pilin engineered with a 19-amino-acid N-t truncation (12). The N-t cysteine tag permitted the covalent attachment of the peptides to a gold electrode and the spontaneous aggregation of the pilins as a conductive monolayer (12). The tight packing of the thiolated pilins on the electrodes clustered the aromatic side chains and recreated the pilus charge hopping pathway (12). The dense packing of the peptides also recreated tunneling regimes through regions of the α-helices lacking aromatic residues and exposed to the solvent the pilin’s C-t random coil that mediates metal binding and reduction at the pilus surface (12, 13). This suggests that N-t truncations can be designed to increase the solubility of the G. sulfurreducens pilin for high-yield recombinant expression of peptides that retain their ability to self-assemble as conductive biomaterials.

Here we describe a strategy for scalable production and purification of recombinant pilins with variable truncations at the N-t (PilA_n_) that reduce their hydrophobicity without perturbing the structural and biochemical motifs critical for self-assembly and conductivity. We also present a protocol for the bottom-up self-assembly of recombinant pilins into protein nanowires having structural and electronic characteristics similar to those of native pili purified from G. sulfurreducens. Unlike the synthesis of inorganic semiconductors, the bottom-up fabrication of pilin-based nanowires does not require complex crystal growth or the use of toxic metals. It relies instead on a hydrophobe-triggered nucleation step and an elongation step that controls the length of the nanowire product. This self-assembly protocol, and the genetic amenability of the recombinant production system, offer opportunities to tune the properties of the peptide, and consequently, the functional characteristics of the resulting nanowire to design novel protein-based conductive nanomaterials for bioelectronics and other applications.

## RESULTS

### Design, recombinant production, and structural characterization of pilin building blocks.

**FIG 2:**
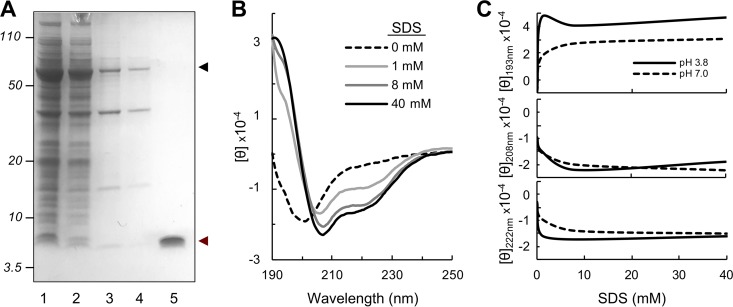
Recombinant production (A) and structural characterization (B and C) of PilA_19_ peptides. (A) SDS-polyacrylamide gel showing the enrichment of the CBD-PilA_19_ fusion protein (black arrowhead) in the soluble protein (lanes 1 and 2) compared to the insoluble protein (lanes 3 and 4) fraction obtained from two independent culture lysates. Lane 5 shows the migration of the recombinant PilA_19_ peptide (red arrowhead) eluted from the chitin column after DTT-induced cleavage from the CBD domain. Numbers to the left of the gel are molecular weight standards in kilodaltons. (B and C) Effect of SDS detergent (B) and pH (C) on PilA_19_ helicity by circular dichroism (CD). Panel B shows CD spectra collected at increasing concentrations of SDS at pH 7. Panel C shows molar ellipticities at key wavelengths in the CD spectra for pH 3.8 and pH 7.

Computational analyses of the PilA sequence via AGGRESCAN (14) identified two regions in the mature pilin peptide (residues 1 to 22 and 25 to 31) as having the highest aggregation propensity (Fig. 1B). Aggregation scores were particularly high for the first 10 amino acids, which were also among the most hydrophobic residues identified in a Kyte-Doolittle plot (15) (Fig. 1B). The aggregation and hydrophobicity analyses therefore predict truncations of the first 21 or 22 amino acids as those having the highest impact on solubility. As this extended truncation preserves the aromatic and charged residues required for fiber formation and conductivity (Fig. 1B), we targeted this N-t region to engineer pilin derivatives suitable for recombinant expression with a self-splicing intein linker and a solubility and affinity tag (chitin binding domain [CBD]). The recombinant approach reproducibly recovered in the soluble fraction of culture lysates fusion proteins containing pilins engineered with truncations of 10, 19, 20, and 22 amino acids (Fig. 1B and C). Affinity chromatography in a chitin column retained the fusion proteins bound to the chitin matrix and permitted the elution of the pilin peptide after inducing the self-splicing of the intein linker with dithiothreitol (DTT) (see Fig. S1 in the supplemental material). Figure 2A shows, as an example, the enrichment of the CBD-PilA_19_ fusion protein in soluble fractions collected from replicate culture lysates and the purification of the PilA_19_ peptide after incubating the chitin-bound CBD-PilA_19_ protein with DTT at room temperature (23°C) for 24 h.

10.1128/mBio.02721-19.1 FIG S1 Effects of temperature and incubation time with dithiothreitol (DTT) on cleavage of the PilA_n_ peptide from the solubility and affinity module (chitin binding domain [CBD]). Addition of DTT to a chitin column containing the fusion protein (CBD-PilA_n_) triggered the self-splicing of the peptide and its elution from the chitin column. After elution, the chitin beads were boiled with 1% SDS to solubilize any matrix-bound proteins (cleaved CBD module and, if present, uncleaved CBD-PilA_n_). The intensity of the CBD over CBD-PilA_n_ protein bands resolved on a 7.5% glycine SDS-polyacrylamide gel was used to estimate the cleavage efficiency. Lanes: no DTT (–) or incubation with DTT (+) for 24, 48, or 72 h. Download FIG S1, TIF file, 0.6 MB. Copyright © 2019 Cosert et al. 2019 Cosert et al. This content is distributed under the terms of the Creative Commons Attribution 4.0 International license.

While the amounts of recombinant fusion proteins recovered from the soluble culture fractions were similar for all the truncated pilins, the amount of peptide eluted from the affinity column after DTT cleavage varied widely (Fig. S1). Cleavage efficiency via intein self-splicing is sensitive to temperature and to the peptide residues adjacent to the intein linker (16). Increasing the temperature from 4°C to 23°C, for example, improved the cleavage efficiency for all the PilA_n_ peptides but also promoted the aggregation of the most hydrophobic peptide (PilA_10_) once cleaved. As a result, the amount of PilA_10_ recovered in solution was too low for visualization in SDS-polyacrylamide gels and required detection via matrix-assisted laser desorption ionization−time of flight (MALDI-TOF) mass spectrometry (Fig. S2). By contrast, truncating 19 amino acids (PilA_19_) permitted the high-yield recovery of the PilA_19_ peptide after cleavage from the CBD tag (Fig. 2A). The 19-amino-acid truncation fused the peptide to the intein linker via a residue (alanine) (Fig. 1B) that is optimal for self-splicing (16). As a result, cleavage efficiency was higher for PilA_19_ than for any other soluble peptides (PilA_20_ and PilA_22_), allowing for the nearly complete recovery of the peptide in the column eluant (Fig. S1). In addition to enabling the highest biosynthetic yields, PilA_19_ retained the helical conformation that is critical for self-assembly and fiber formation once in the presence of a hydrophobe. We demonstrated this by investigating the folding dynamics of the PilA_19_ by circular dichroism (CD) as a function of the concentration of a detergent such as SDS (Fig. 2B). The far UV CD spectrum of the peptide in 10 mM potassium acetate buffer at pH 7 showed very low ellipticity above 210 nm and a strong negative signal around 200 nm, consistent with a disordered peptide (17). However, the addition of SDS shifted the CD spectrum and revealed the characteristic maxima (at ∼190 nm) and minima (at ∼208 and ∼222 nm) of α-helical conformations (18). Furthermore, the intensity of the positive (190 nm) and negative (208 nm and 222 nm) helical signals reached maxima at or above the critical micellar concentration (CMC) of the detergent (∼8 mM in water) (19, 20) (Fig. 2C). At this threshold concentration, SDS recreates the hydrophobic environment of the inner membrane (21), where the pilins are stored prior to assembly to stabilize their α-helical conformation (6). Concentrations at or above the CMC (8 mM and 40 mM) also produced intensity ratios of 222 nm over 208 nm (∼0.7) close to the 0.8 ratio expected for a single-stranded α-helix (22). As the peptide has a negative net charge of −1.1 at neutral pH, we minimized electrostatic effects with SDS by collecting CD spectra of control solutions at a pH of 3.8. At this acidic pH, below the theoretical isoelectric point (pI, 4.86) of PilA_19_, the peptide has a net positive charge of +3.2 that cancels out electrostatic repulsion forces with the anionic detergent. As predicted, the intensity of the α-helical signature peaks was greater at pH 3.8 than at pH 7 (Fig. 2C). The more favorable electrostatic interactions between the peptide and detergent at the acidic pH also increased the pilin’s helical content (from ∼45% to 56% at or above the CMC and from 27% to ∼49% below the CMC). The higher helical content of the PilA_19_ peptide at the acidic pH also produced 222-nm/208-nm intensity ratios of about 1, consistent with the assembly of two or more α-helices in coiled-coil configurations (22). These results demonstrate that PilA_19_ can adopt the helical conformation that is critical for pilin self-assembly and electronic coupling in the pilus fiber. Additionally, the studies highlighted the critical role that hydrophobicity and electrostatics have in modulating the folding and self-assembly of the peptides.

10.1128/mBio.02721-19.2 FIG S2 MALDI-TOF mass spectrometric (MS) analysis of PilA_n_ peptides eluted from the chitin column after cleavage with DTT for 24 h at 23°C. The theoretical molecular masses of the truncated pilins are as follows: 5,431 Da (PilA_10_), 4,595 Da (PilA_19_), 4,524 Da (PilA_20_), and 4,314 Da (PilA_22_). Download FIG S2, TIF file, 0.3 MB. Copyright © 2019 Cosert et al. 2019 Cosert et al. This content is distributed under the terms of the Creative Commons Attribution 4.0 International license.

### Fiber formation via self-assembly of PilA_19_ peptides.

**FIG 3:**
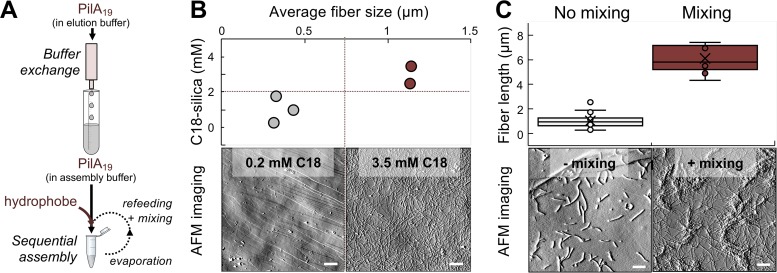
Evaporation-induced self-assembly of recombinant PilA_19_ peptides in the presence of a hydrophobe. (A) Protocol illustrating the key steps of hydrophobe-triggered assembly of PilA_19_ as fibers. (B) Hydrophobe (C18-silica particles) dose effect on fiber formation, estimated as average fiber size by dynamic light scattering (plot [top]). Bottom panels show AFM images of samples from 0.2 or 3.5 mM C18-silica reaction mixes after deposition on highly oriented pyrolytic graphite (HOPG). (C) Effect of reaction mixing on fiber elongation (box plot). Boxes in plot contain 50% of all values, and whiskers represent the 25th and 75th percentiles of fiber lengths measured by analyzing with the ImageJ software AFM images of random HOPG fields with samples (bottom). The median value is shown as a horizontal line across the boxes, the average value is shown as a cross, and outliers are shown as circles outside the boxes. The scale bar in the AFM images in panels B and C is 1 μm.

The finding that hydrophobicity induces the helical folding of PilA_19_ and subunit assembly in solution prompted us to investigate hydrophobic conditions optimal for controlled self-assembly of the PilA_19_ peptides into fibers. Figure 3A shows the main steps of a protocol optimized for bottom-up fabrication of PilA_19_ fibers. The fabrication starts with a buffer exchange step that resuspends the peptides in a buffer of acetonitrile and methanol suitable for evaporation-induced self-assembly in the presence of a hydrophobe. Acetonitrile has lower polarity than water to help maintain recombinant pilin peptides in solution (12). Methanol, on the other hand, stabilizes the helical conformation of peptides while in solution (23). Addition of a hydrophobe triggered self-assembly of the peptides, as reported for other bacterial pilins (24–26), whereas controlled evaporation of the solvent increased molecular crowding and facilitated peptide-peptide interactions needed for fiber formation (Fig. 3A). Octadecane, a straight-chain alkane hydrocarbon of 18 carbon atoms (C18), was a suitable hydrophobe to promote the nucleation of the pilins and fiber formation (Fig. S3). Atomic force microscopy (AFM) imaging of the octadecane-triggered assembly reaction revealed, however, an extensive coating of the fibers and underlying electrode with a hydrocarbon layer that prevented conductivity measurements (Fig. S3). To bypass this limitation, we replaced octadecane with silica particles coated with octadecyl carbon chains (C18) (Fig. 3). By conducting the buffer exchange step in a reverse-phase column packed with a reverse-phase resin of C18-silica particles (55 to 105 μm in diameter), we simultaneously eluted the PilA_19_ peptide and C18-silica nanoparticles approximately 25 to 50 nm in diameter that were optimal for hydrophobe-triggered fiber formation (Fig. S4).

10.1128/mBio.02721-19.3 FIG S3 Octadecane-triggered formation of PilA_19_ fibers. (A) UV-vis spectrum of a pilin solution eluted in assembly buffer from an Oasis Max exchange cartridge showing the absorbance peak of DTT at 205 nm (inset, DTT standard curve). (B) Tapping mode AFM showing PilA_19_ fibers formed after evaporation-induced assembly in the presence of octadecane (1:100 aqueous solution) but not in the absence of octadecane (1:100 aqueous solution). Controls without PilA_19_ monomers show column resin residues (–octadecane) and a hydrocarbon layer (+ octadecane) on the electrode surface. Scale bar, 1 μm. Download FIG S3, TIF file, 1.0 MB. Copyright © 2019 Cosert et al. 2019 Cosert et al. This content is distributed under the terms of the Creative Commons Attribution 4.0 International license.

10.1128/mBio.02721-19.4 FIG S4 Effect of C18 column wash on nucleator concentration. (A) UV-vis spectra of eluants from triplicate C18 columns washed with 5 ml (dark color; optimal protocol for PilA_19_ fiber formation) or 18 ml (light color; extended wash preventing PilA19 fiber formation) of ddH_2_O prior to buffer exchange with assembly buffer. Reduced washes produce the spectral peaks for DTT (∼205 nm) and silica nanoparticles (∼245 nm). Inset shows an AFM image (tapping mode) of C18-silica particles (25 to 50 nm in diameter) eluting from the column after a 5-ml wash. (B and C) Standard curves of silica absorption at 245 nm in solutions made with assembly buffer and the column’s C18-silica resin (B) or pure silica particles of sizes similar to those of the column’s resin (63 to 200 μm) or the eluted particles (0.5 to 1 μm) (C). Download FIG S4, TIF file, 0.1 MB. Copyright © 2019 Cosert et al. 2019 Cosert et al. This content is distributed under the terms of the Creative Commons Attribution 4.0 International license.

We gained insights into the rate-limiting steps of PilA_19_ assembly by investigating the effect of hydrophobe concentration in fiber formation. For these experiments, we estimated fiber size in reactions with different concentrations of the C18-silica particles by dynamic light scattering (DLS) and used an AFM to image the structural features of the assemblies after sample deposition on the surface of a freshly cleaved highly oriented pyrolytic graphite (HOPG) (Fig. 3B). C18-silica particle concentrations of ∼3.5 mM triggered fiber formation (Fig. 3B) and supported maximum assembly efficiencies (40 to 55% of the pilin monomers assembled as fibers). Efficient assembly also required reaction mixing. Unmixed assembly reaction mixtures containing the same concentration of the nucleating C18-silica particles produced fibers with average lengths of 1 (±0.5) μm and reduced assembly efficiencies to 14% (Fig. 3C). However, mixing the assembly reactions by aspiration with a micropipette during the peptide refeeding steps promoted the assembly of the pilins and the growth of fibers 6 (±1) μm long (Fig. 3C). This suggests that the initial evaporation step of a 1-ml volume of assembly buffer with the peptide (∼3 mg) stimulated pilin nucleation by the C18-silica hydrophobe, whereas mixing during the four sequential peptide refeeding steps (total of 6 mg of peptide) increased the number of nucleation sites available for peptide assembly and the availability of peptide building blocks to grow the fibers. For controls, we also conducted assembly reactions with mixing in the presence of suboptimal concentrations of the hydrophobe (0.2 mM). The limited availability of the nucleating C18-silica particles in the reactions led to the formation of small aggregates interspersed with short fibers (Fig. 3B). Thus, fiber elongation is both dependent on hydrophobe concentration and reaction mixing. Additional refeeding/mixing did not change the kinetics of fiber formation, suggesting that an equilibrium between free peptide and fibers had been reached that prevented new nucleation and elongation reactions.

### Biochemical and electronic characterization of PilA_19_ fibers.

The optimized hydrophobe-triggered assembly protocol, with sequential refeeding and mixing steps, consistently produced long, flexible fibers. The average diameter of the PilA_19_ fibers (calculated as AFM height) was ∼2 nm, as reported for the native PilA pilus fibers (8). AFM images of the PilA_19_ fibers also revealed some supramolecular structures (braids of two fibers), but most of the fibers were present as well-dispersed filaments (Fig. S5A). This contrasts with the extensive supramolecular aggregation of native pili, which persists even after minimizing the pili’s surface electrostatics in alkaline buffers (2, 8). The good dispersion of the PilA_19_ fibers permitted the collection of CD spectra (Fig. S5B) similar to the CD profiles of other bacteria type IVa pili (27). From the CD spectrum, we calculated an intensity ratio of 222-nm absorbance over 208-nm absorbance of 0.75 for the PilA_19_ fibers, which is close to the ∼0.8 intensity ratios that result from the α-helical conformation of the peptide monomers (22). In contrast, the CD spectra of control solutions with the native pili were convoluted by numerous peaks (Fig. S5C) and had intensity ratios at 222 and 208 nm of ∼1, as reported for supramolecular assemblies (22). This complex spectral profile results from the random aggregation of the native pilus fibers, which form thick bundles that can only be destabilized with strong denaturants such as urea (Fig. S5C). The aggregative nature of the native pili is the result of surface electrostatics as well as fiber length, which can be greater and more heterogenous than in the samples containing the approximately 6-μm-long PilA_19_ fibers.

10.1128/mBio.02721-19.5 FIG S5 Biochemical characterization of PilA_19_ fibers. (A) AFM imaging of PilA_19_ fibers assembled *in vitro*. Inset shows single fibers and braided supramolecular structures with line scans used to determine the AFM height (red, single fiber; blue, braided fibers). Scale bar, 0.2 μm. (B and C) CD spectra of PilA_19_ fibers (B) and native PilA pili untreated (solid line) or treated with 8 M urea (dashed plot) (C). Download FIG S5, TIF file, 0.9 MB. Copyright © 2019 Cosert et al. 2019 Cosert et al. This content is distributed under the terms of the Creative Commons Attribution 4.0 International license.

**FIG 4:**
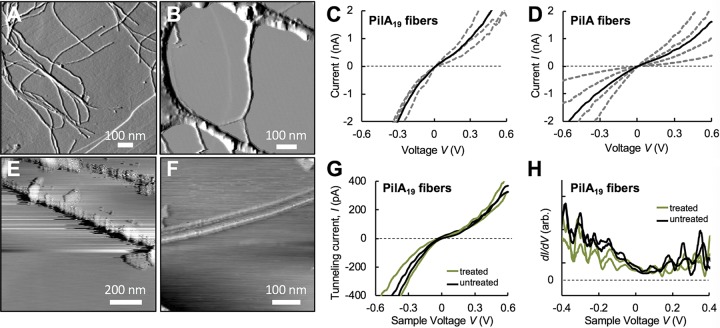
Electronic characterization of PilA_19_ pili. (A and B) AFM amplitude image of PilA_19_ (A) and native PilA (B) fibers deposited on HOPG, showing the dispersion of the PilA_19_ fibers and extensive supramolecular aggregation of the native pili. (C and D) Representative current-voltage (*I*-*V*) curves of PilA_19_ and PilA fibers by conductive probe AFM (individual measurements are shown as dashed lines, and the average of the four measurements is shown as a solid black line). (E and F) Room temperature scanning tunneling microscopy (STM) topographic image of untreated (E) or chemically fixed (F) PilA_19_ fibers (0.5 V, 350 pA). (G) Average current-voltage (*I-V*) STM tunneling spectra of two sequential measurements for each of two pilus regions in untreated (black) and fixed (green) samples. (H). Differential conductance (*dI/dV*) curves of the untreated and chemically treated PilA_19_ fibers, calculated as the numerical derivative of the *I-V* curves in panel G.

The dispersion of the PilA_19_ fibers in aqueous media also facilitated AFM imaging of individual filaments after deposition onto HOPG (Fig. 4A). Control samples with the native G. sulfurreducens pili (denoted PilA fibers), on the other hand, showed extensive supramolecular aggregation that made the identification of single filaments especially laborious (Fig. 4B). Additionally, the reduced aggregative nature of the PilA_19_ fibers improved electrical contact with the underlying electrode. As a result, conductive probe AFM (CP-AFM) measurements of the transversal current flowing through different fibers while sweeping the applied voltage (*I-V*) were less variable than with PilA fibers (Fig. 4C). Average *I-V* curves from four independent PilA_19_ or PilA fibers were, however, similar (Fig. 4C and D). Furthermore, the average resistance of the PilA_19_ fibers (∼900 MOhms at ±100 mV) was within the order of magnitude calculated for the native wires (∼925 MOhms). In addition, *I-V* curves collected for the PilA_19_ and PilA fibers by CP-AFM were similarly asymmetric, showing a rectification behavior such that more current was measured at negative voltages than at the same positive voltages (Fig. 4C and D). Thus, the average rectification score (calculated as current at positive over negative voltage) for the PilA_19_ fibers was ∼0.5 and 0.7 at biological (±100 mV) and higher (±600 mV) voltages. Similarly, the PilA fibers had rectification scores below 1 (∼0.7) at both. Thus, current flow through the pilus is more efficient from the electrode to the AFM tip (more current produced at negative voltages), which is also the biological path for the discharge of respiratory electrons from charged electron carriers in the cell envelope to the pilus and then to extracellular electron acceptors.

We used scanning tunneling microscopy (STM) to characterize nanoscale spatial variations in electronic properties within individual PilA_19_ fibers (Fig. 4E). The higher spatial resolution of the STM technique compared to CP-AFM resolved beadlike structural features in hydrated PilA_19_ fibers previously described for the native PilA pili (28). The bright spots are regions of the fiber with higher local electronic density of states and, thus, regions that supply more tunneling current (28). The molecular substructures identified in the PilA_19_ fibers have periodicities that match well with those reported for the grooves and ridges that form the surface landscape of the native pili (28). The STM diameter estimated for the PilA_19_ fibers (∼5 to 7 nm) was also within the ranges reported for the native PilA filaments prior to deconvoluting for the broadening tunneling effect caused by the tip when scanning a nanowire (8, 28). Also as reported previously for the PilA filaments (28), STM imaging of the PilA_19_ fibers improved with a chemical fixation step (Fig. 4F). The chemical treatment immobilized more fibers on the surface and improved electrical contact with the electrode. As a result, the interactions between the STM tip and the biomaterial were more stable when probing chemically fixed PilA_19_ fibers, producing cleaner STM topographic images (Fig. 4F). Importantly, chemical fixation did not substantially affect the measured conductivity, as indicated by the overlapping *I*-*V* curves collected when probing fixed locations of untreated (hydrated) and chemically treated PilA_19_ fibers while sweeping the voltage at ±600 mV (Fig. 4G). Additionally, the STM *I-V* curves of untreated and treated fibers reproduced the ohmic response of the biomaterial in the ±100 mV biological voltage range observed by CP-AFM and had similar slopes, thus a similar resistance to the passage of electrons. Plots of the differential conductance (*dI*/*dV*) of the untreated and treated PilA_19_ fibers versus the tip sample bias voltage (*V*) confirmed these similarities and revealed electronic states at low voltages that never reach zero conductance (Fig. 4H), a distinctive electronic feature of the native PilA pili that results from a very small electron band gap (8, 28). The STM differential conductance plots also confirmed the asymmetric conductance reported for native pili (8, 28), as expected for a biomaterial that favors current flow from negative to positive voltages, even at the low voltages (i.e., ±100 mV) that drive the flow of respiratory electrons through the pili and onto the iron oxides (29).

## DISCUSSION

The recombinant production of peptides derived from the conductive pilin of G. sulfurreducens permitted the synthesis at high yields of a soluble pilin peptide (PilA_19_) carrying an N-t truncation of the first 19 amino acids of the mature PilA pilin. The truncation removed hydrophobic amino acids at the N-t that are known to participate in biological assembly processes (e.g., F1 and E5) (7, 10) (Fig. 1), but the peptide retained the α-helical conformation that is needed for pilin-pilin hydrophobic interactions and self-assembly (Fig. 2). Importantly, the PilA_19_ truncation preserved the charged residues of the pilin that MD simulations predict to form salt bridges between neighboring pilins (Fig. 1), establishing intermolecular bonds critical to the structural integrity of the fiber core and the electronic coupling of aromatic side chains (5, 8). Supporting the computational predictions, the recombinant PilA_19_ peptide self-assembled into a conductive fiber that exhibited biochemical, structural, and electronic properties similar to those of a native PilA pilus (Fig. 3 and 4).

**FIG 5:**
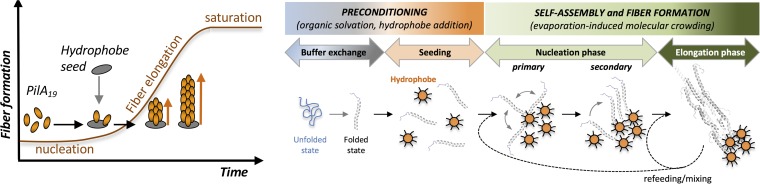
Stepwise formation of PilA_19_ fibers. (Left) Nucleation-dependent polymerization model of hydrophobe-triggered pilin assembly showing the three phases of nucleation, fiber elongation, and saturation. (Right) Steps in the bottom-up fabrication of protein nanowires using PilA_19_ peptide building blocks and a hydrophobe.

Similarly to amyloid peptides, the pilins polymerized *in vitro* following kinetics that fit the typical nucleation-dependent polymerization model (30) (Fig. 5). Nucleation-controlled aggregation kinetics include an initial lag phase of molecular organization and peptide nucleation that can be accelerated in the presence of seed molecules. A linear phase of fiber growth then follows until reaching a saturation or stationary phase, which marks the equilibrium between soluble monomers and fibers and the end of fiber growth. As shown in Fig. 5, we minimized the lag phase by suspending the PilA_19_ monomers in an organic solvent that stabilized the peptide’s helical conformation and added a hydrophobe to seed pilin nucleation and guide fiber growth. The controlled evaporation of the solvent increased molecular crowding and promoted the initial nucleation of the pilins on the seed molecules (primary nucleation) and their spontaneous self-assembly as short fibers (secondary nucleation). Fiber elongation depended on the availability of hydrophobe molecules and pilin building blocks supplied in subsequent refeeding steps. It was also sensitive to reaction mixing, which increased the number of nucleation sites and the incorporation of monomers into the growing fibers until reaching the saturation phase. Mechanical fragmentation of preformed fibers may have also contributed to increasing the number of nucleation sites, as reported for amyloid fibers (31) and peptide nanotubes (32).

Octadecane, whether in solution (see Fig. S3 in the supplemental material) or immobilized on silica particles (Fig. 3), was a suitable hydrophobe to trigger pilin nucleation and fiber formation. Thus, the hydrophobe does not need to be incorporated into the fiber and can be provided as nanoparticles to nucleate the pilins into fibers (Fig. 5). The addition of the hydrophobe in a surface-constrained form has additional advantages. The particle size controls the curvature and surface area of the substrate and the contact effects that modulate the rate of nucleation. Surface hydrophobicity impacts the effectiveness of seed particles in fibril formation (33) and could be controlled with the type of alkane coating (34) or with the use of nanoparticle materials with higher water contact angle than silica (33). Hydrophobe-bound silica nanoparticles also permitted their separation from the fibers after acetone precipitation of the proteins at the end of the assembly reaction. Moreover, the emission of silica from nanosized particles in a precise region of the UV spectrum (Fig. S4) proved useful to measure the concentration of hydrophobe. At optimal hydrophobe concentrations and with sufficient peptide refeeding and reaction mixing steps, we synthesized PilA_19_ fibers approximately 6 μm long that dispersed well in mild aqueous solutions (Fig. 3). This contrasts with purification protocols available for native pili (2, 8), whose longer and heterogeneous length promotes the formation of large supramolecular structures that are difficult to disrupt without denaturing the pilus fiber core (Fig. 3). The reduced aggregation of the PilA_19_ fibers also facilitated their deposition on electrode surfaces and electronic characterization by scanning probe methods (Fig. 4). Reproducible electronic probing of hydrated fibers by CP-AFM is challenging because it affects the local electrical contact with the underlying electrode and, in turn, the measured conductivity. Supramolecular aggregation in the native PilA pili enhances these effects and the variability of the conductivity measurements compared to the more dispersed PilA_19_ fibers (Fig. 4). Despite these differences, scanning probe methods calculated a similar average electrical resistance for the PilA and PilA_19_ fibers (900 to 925 MOhms by CP-AFM) and revealed the characteristic topographic periodicities (roughly every 10 nm by STM) that arise from the conserved helical arraignment of the pilins (28). We reduced the contact resistance between the fibers and the underlying electrode and, therefore, sample-to-sample variability through chemical fixation (Fig. 4). Importantly, the chemical treatment did not affect the conductive properties of the fibers, a property that can facilitate the integration of the protein nanowires with inorganic nanomaterials in electronic devices. Also important for applications in bioelectronics are the rectifying properties of the PilA_19_ fibers, which like the native PilA pili, transport charges more efficiently from negative to positive voltages (Fig. 4). Rectification could reflect mechanistic differences in the directionality of charge transport through the pilus that favor the biological flow of electrons from the cell envelope to the pilus-bound electron acceptor (12). Surface effects also need to be considered that reflect differences in the rates of charge injection from the large electrode surface to the pilus compared to the reverse flow of electrons from the much smaller conductive tip positioned on top of the fiber. Tip-pilus interactions are also likely to be influenced by the net negative charge of the fiber surface (5), which could limit charge injection, and thus current flow, at positive bias voltages. The charge of the pili is less critical when electrons are injected from the underlying electrode into the fiber, as the fiber’s electrical contact can involve positively charged or noncharged pilus regions.

The demonstration that truncated, conductive type IV pilins can be expressed in heterologous hosts, purified, and then self-assembled *in vitro* to form fibers having an electrical conductivity comparable to the conductivity of the native pili confirms unequivocally that a peptide assembly can conduct electrons in the absence of metals and/or organic redox cofactors, as demonstrated for the native PilA pili (8). The production of conductive pili with recombinant peptides also establishes a versatile new platform for bottom-up fabrication of nanowires that leverages powerful, synergistic tools for customizing the properties of the biomaterial. These tools include advanced computational simulations (5) to accurately predict the impact of genetic changes on the physical, chemical, and electrical properties of the nanowire product and well-established genetic engineering tools to introduce these changes in the pilin’s amino acid sequence prior to their recombinant production in scalable, high-density E. coli fermentations. MD models such as the one that guided our pilin designs (Fig. 1) can accurately simulate the pilin’s helicity, the spatial arrangement of the pilins within the helical fiber, and the way in which the proximity of charged and aromatic amino acids establishes low-resistance paths for electron conduction through the fiber (5). The predictive power of MD simulations expedites progress via *in silico* testing of hypotheses about how genetic changes could be used to improve the fibers’ properties or even add novel functionalities. Given the sensitivity of pilus charge transport to the number of aromatic contacts formed along the fiber or the local electrostatics around the aromatic side chains (8), amino acid substitutions and alignment could be modified to control the fibers’ electrical or biochemical properties. Adding functional groups suitable for click chemistries is also possible. The lack of cysteines in the pilin’s amino acid sequence makes this amino acid a suitable functional tag for attachment to metal electrodes (12, 13). This and other functional tags could provide chemical control of deposition of protein nanowires in precise orientation in electronic devices. In addition, the ease with which aqueous solutions containing pilin monomers or oligomers could be processed would facilitate further chemical, thermal, or mechanical processing. Many of these strategies could also be used to modulate the fibers’ rectifying behavior and fabricate protein diodes for incorporation into lattices and heterostructures on inexpensive materials suitable for energy applications (35). However, unlike chemically synthesized peptides, the recombinant production of pilin monomers and assembly into protein nanowires is scalable, simultaneously addressing the interrelated challenges of sustainable supply, engineering, and production in the electronics industry (36).

Length control is perhaps one of the most difficult parameters to standardize in self-assembly processes of peptides in solution (32). However, it was possible to modulate the length of the PilA_19_ fibers by introducing refeeding steps of peptide building blocks with gentle reaction mixing (Fig. 3). A scaled-up nanowire production process with mechanical agitation could minimize spatial chemical gradients and ensure consistent self-assembly dynamics for reproducible fabrication of nanowires having a desired length in addition to other properties. For example, the ability to control nanowire length and orientation would be desirable for growing nanowires as conductive bridges between electrodes. Also, the ability to control nucleation density and fiber elongation kinetics on an electrode’s surface would be desirable for fabricating conductive nanowire brushes for energy storage or sensing (37). Near-term applications enabled by the conductive pilin platform could harness the ability of the pilus surface ligands and structural motifs to selectively bind, and reductively precipitate, divalent cations such as the uranyl (2) and cobalt (13) cations. The nucleation-controlled aggregation kinetics of the pilins in the presence of a seed molecule affords opportunities to nucleate the fibers in a high-density spatial array and to control their length by adjusting the supply of peptide building blocks. The potentially low cost, high binding affinity, and extremely high surface area of the resulting nanobrush electrodes could provide inexpensive and highly effective platforms for the recovery of water-soluble cations of toxic, radioactive, rare, or precious metals, such as those now abundant in electronic waste (38, 39).

## MATERIALS AND METHODS

### Bacterial strains and culture conditions.

Geobacter sulfurreducens strain PCA was routinely grown in anaerobic NB medium (40) with 20 mM acetate as the electron donor and 40 mM fumarate as the electron acceptor. Genomic DNA extracted from these cultures was used as the template to PCR amplify the native *pilA* gene (GSU1496) and engineer recombinant pilin production systems in E. coli Rosetta 2 (DE3)/pLysS cells (Novagen) as described below. The E. coli cultures were propagated in Luria-Bertani (LB) medium supplemented with antibiotics and preserved in 20% glycerol at –80°C.

### Design, recombinant production, and purification of truncated pilins.

The truncated pilins used in this study (PilA_10_, PilA_19_, PilA_20_, and PilA_22_) are derivatives of the mature PilA peptide of G. sulfurreducens (i.e., the pilin without its signal peptide) carrying 10, 19, 20 and 22 amino acid truncations at the peptide’s N terminus (N-t). The truncation design was based on analyses of the hydrophobic regions and aggregation potential of the PilA peptide based on computational predictions with AGGRESCAN (14), grand average hydropathy (GRAVY) scoring (15), and Kyte-Doolittle test (15). All truncations preserved the aromatic and charged amino acids of the pilin that are critical for conductivity and formation of salt bridges (5). Pilin design also involved computational analyses of molecular dynamics (MD)-optimized structural models of the PilA pilus fiber (GPIL-WT.pdb) and PilA pilin (pilin-WT.pdb) (5) constructed with the MacPyMOL: PyMOL v1.8.2.2 software enhanced for Mac OS X (Schrödinger LLC). Pilin truncations were introduced with PCR primers (see Table S1 in the supplemental material) using as the template the *pilA* gene (GSU1496) of G. sulfurreducens cloned in the pTYB11 plasmid vector (IMPACT-CN system; New England Biolabs). The resulting plasmids (pTYB11*::pilAn*, where *n* stands for the number of N-t amino acids truncated) were transformed into E. coli Rosetta 2 (DE3)/pLysS cells (Novagen) for recombinant expression of the PilA_n_ peptide fused at the N-t to an intein linker and a chitin binding domain (CBD), as described elsewhere (12).

10.1128/mBio.02721-19.6 TABLE S1 Primers (forward and reverse) used to clone the mature *pilA* sequence in the expression vector pTYB11 and PCR-amplified truncated derivatives (*pilA_n_*). Download Table S1, DOCX file, 0.02 MB. Copyright © 2019 Cosert et al. 2019 Cosert et al. This content is distributed under the terms of the Creative Commons Attribution 4.0 International license.

Recombinant expression of CBD-PilA_n_ fusion proteins in the E. coli host was studied in 1-liter cultures of LB broth supplemented with 100 μg/ml ampicillin and 20 μg/ml chloramphenicol and incubated at 37°C to an optical density at 600 nm (OD_600_) of ∼0.4 before induction with 50 mM isopropyl-β-d-1-thiogalactopyranoside (IPTG) during overnight incubation at 16°C as previously described (13). Cells harvested by centrifugation (4,000 × *g* for 10 min) were resuspended in 20 mM Tris-HCl buffer (100 mM NaCl, 1 mM EDTA, 1% 3-[l(3-cholamidopropyl)-dimethylammonio]-1-propanesulfonate (CHAPS)) and lysed by tip sonication. Centrifugation of the lysate (12,000 × *g* for 30 min at 4°C) separated the soluble proteins, including the fusion protein, in the supernatant fraction. Purification of the fusion protein from the other soluble proteins was by affinity chromatography in a chitin column (New England Biolabs; ca. 40-ml bed volume) equilibrated with 200 ml of column buffer (20 mM Tris, 100 mM NaCl, 1 mM EDTA [pH 7.4]). After incubation with the soluble protein fraction at room temperature for 20 min to promote the attachment of the fusion protein to the chitin matrix, we washed the column with 200 ml of buffer at increasing salt concentrations (20 mM Tris, 1 mM EDTA, 0.6 to 1 M NaCl [pH 7.4]) to remove the unbound proteins.

Cleavage of the PilA_n_ peptides from the chitin-bound fusion protein was by induction of intein self-splicing with 50 mM 1,4-dithiothreitol (DTT). To do this, we incubated the column with ∼200 ml of cleavage buffer (20 mM Tris, 100 mM NaCl, 50 mM DTT [pH 9]) for 24, 48, or 72 h at room temperature (23°C) or, when indicated, at 4°C to minimize the aggregation of the peptide after cleavage. A column wash with the same buffer but without DTT (elution buffer) eluted the PilA_n_ peptides in 2-ml eluent fractions, which we identified by their absorbance at 280 nm. Because some of the peptides aggregated during elution, cleavage efficiency was estimated from the ratio of the CBD over the full CBD-PilA_n_ proteins that remained bound to the chitin column after DTT cleavage and elution of the peptide. To do this, we removed the chitin beads from the column and resolubilized the chitin-bound proteins in 1% sodium dodecyl sulfate (SDS) at 100°C. Separation of the solubilized proteins was in a 7.5% Tris-glycine SDS-polyacrylamide (Bio-Rad) run for 30 min at 200 V in Tris-glycine-SDS buffer (25 mM Tris, 192 mM glycine, 0.1% [wt/vol] SDS) using a Bio-Rad Mini Trans-Blot cell system. The proteins in the gels were stained with Bio-safe Coomassie (Bio-Rad, Hercules, CA) for 1 h and destained in double-distilled water (ddH_2_O). The densities of the fast-migrating band (CBD module) and the slower-migrating CBD-PilA_n_ protein band were used to calculate the percentage of CBD-PilA_n_ that was cleaved.

Cleavage at room temperature for 24 h was optimal for high-yield recovery of PilA_19_ and was used thereafter. Peptide-containing fractions were pooled before estimating the protein concentration by absorbance at 280 nm using a NanoDrop spectrophotometer (Thermo Scientific). SDS-polyacrylamide gel electrophoresis (PAGE) was used to monitor the recombinant expression of the fusion protein (CBD-PilA_n_) and purity of the PilA_n_ peptide eluting from the chitin column using 10 to 20% Tris-Tricine polyacrylamide gels run for 75 to 120 min at 100 V in Tris-Tricine-SDS buffer (100 mM Tris, 100 mM Tricine, 0.1% [wt/vol] SDS). Proteins in the Tricine gels were fixed for 30 min with an aqueous solution of 50% methanol and 40% acetic acid prior to staining with Bio-safe Coomassie (Bio-Rad, Hercules, CA) for 1 h and destaining with ddH_2_O until bands were visible. The mass of the peptide was confirmed by matrix-assisted laser desorption ionization−time of flight (MALDI-TOF) mass spectrometry using a 1-μl peptide solution mixed with 1 μl of 50 mM 3,5-dimethoxy-4-hydroxycinnamic acid in 50% acetonitrile (CH_3_CN)−0.5% trifluoroacetic acid (TFA) and dried on a sample plate. Mass spectrum collection was on a time of flight (TOF) Voyager-DE Pro-MALDI-TOF mass spectrometer (Applied Biosystems, Framingham, MA).

### Circular dichroism.

PilA_19_ peptides purified in elution buffer were dialyzed against 10 mM potassium acetate buffer (with 50 mM Na_2_SO_4_ [pH 3.8]) using Spectra/Por Biotech cellulose ester dialysis membranes (molecular weight cutoff [MWCO] of 100 to 500 Da). The peptide concentration was determined from the difference spectrum (320 to 270 nm) of the protein dissolved in 1 ml of 6 M guanidine hydrochloride at pH 12.5 versus pH 7.1 (41) and with the known molar extinction coefficients of tyrosine and tryptophan residues (42) using the following equation:

(1) $$C=\frac{A_{293}}{2,357Y+830W}$$

where *A*_293_ is the absorbance at 293 nm in the difference spectrum, *Y* is the number of tyrosines (3 in PilA_19_), and *W* is the number of tryptophan residues (0 in PilA_19_).

The concentration of the peptide in the buffer was adjusted to approximately 50 μg/ml prior to circular dichroism (CD) spectroscopy. When indicated, SDS was added to the peptide solution at a final concentration of 1, 8, or 40 mM. The peptide solutions were dispensed in a quartz cuvette (0.1-cm path length) (Starna Cells Inc.), and their CD spectra in the 190- to 360-nm range were collected at 0.5-nm increments (5-s integration time) using a Chirascan spectrometer (Applied Photophysics Ltd., Leatherhead, United Kingdom). The spectra were baseline corrected and smoothed using a third-order Savitsky-Golay filter. The CD instrument units (θ, millidegrees) were converted into mean residue molar ellipticity [θ] units using the Wallace and Janes equation (43):

(2) $$\left[\text{θ}\right]=\left(\frac{\text{θ}\times 0.1\times \text{MRW}}{c\times l}\right)$$

where *c* is the peptide concentration in milligrams per milliliter, *l* is the path length of the cuvette in centimeters (0.1 cm), and MRW is the mean residue weight of the sample estimated from the molecular mass (MW) in daltons (4,524 Da for PilA_19_) and the number *n* of amino acid residues (42 for PilA_19_), as follows:

(3) $$\text{MRW}=\frac{\text{MW}}{n-1}$$

The CD data were used to estimate the α-helix content of peptide using the program CONTINLL at the DICROWEB server (44, 45). The program is a modification of the CONTIN method (46, 47) that uses a ridge regression algorithm to estimate the CD spectra of unknown proteins by comparison to a linear combination of CD spectra of *N* reference proteins with known conformations (41, 48). Because the reference proteins are predominantly globular, the conformation estimates for peptides, fibrous proteins, and membrane proteins are approximate (41, 43, 44). The program evaluates the goodness of fit parameter normalized mean residue standard deviation (NMRSD), which is defined as follows:

(4) $$\text{NRMSD}={\left[\frac{\sum {\left({\text{θ}}_{\exp}-{\text{θ}}_{\text{cal}}\right)}^{2}}{\sum {\left({\text{θ}}_{\exp}\right)}^{2}}\right]}^{1/2}$$

where θ_exp_ and θ_cal_ are the experimental and calculated ellipticity values at a specific wavelength. An NRMSD value of less than 0.1 is generally considered a good fit (43). Thus, NRMSD values above 0.1 were rejected.

The CD spectra were also collected for fibers assembled with recombinant PilA_19_ peptides resuspended for 30 min in 10 mM potassium acetate with 50 mM Na_2_SO_4_ (pH 7). A 500-μl aliquot of 40 μg/ml (Nanodrop estimate) of the pilus solution was dispensed into a quartz cuvette with a 1-mm path length (Starna Cells Inc., Atascadero, CA) and scanned from 190 to 260 nm at 0.5-nm increments with a 5-s integration time with automated baseline subtraction. Native pili, purified from G. sulfurreducens as described elsewhere (2), were used as controls before or after denaturation with 8 M urea. All the scans were adjusted from θ, millidegrees, to molar ellipticity using equation 2 as described above.

### *In vitro* assembly of PilA_19_ pilins.

Synthesis of PilA_19_ fibers followed a protocol that incorporated a buffer exchange step to resuspend the peptides in assembly buffer (80:20, acetonitrile-methanol) and an evaporation-induced assembly in the presence of a hydrophobe. The standard protocol, optimized for maximum PilA_19_ assembly efficiency of ∼50%, used a reverse-phase C18 column (Sep-Pak C18 3 cc Vac Cartridge, 55 to 105 μm particle size; Waters Corporation, Milford, MA) for buffer exchange inside an anaerobic chamber (COY Labs). The resin in the cartridges was first hydrated with 5 ml of acetonitrile and equilibrated with 5 ml of ddH_2_O, following the manufacturer’s recommendations. A solution of the recombinant peptide (8-9 mg of PilA_19_ in ∼10 ml of elution buffer) was applied to the column by gravity flow, and the peptide retained in the C18 resin was washed with 5 ml of ddH_2_O before elution in a disposable glass tube with 3 ml of a freshly prepared assembly buffer. When indicated, the washing step was extended from 5 to 9, 12, 15, or 18 ml of ddH_2_O. DTT and C18-silica particles (straight-chain alkane hydrocarbon of 18 carbon atoms [C18]-silica particles) coeluting with the peptide were identified in the UV-visible (UV-vis) spectrum of the solution as absorbance peaks at 205 nm (DTT) and 245 nm (silica) using a Shimadzu UV-2401PC spectrophotometer. Control solutions with pure silica particles demonstrated the sensitivity of the detection method to particle sizes of less than 1 μm (Fig. S4). Thus, we used standard solutions of pure silica particles 0.5 to 1 μm in diameter to calculate the concentration of C18-silica particles in the solution.

When indicated, the buffer exchange step was conducted in an Oasis Max extraction cartridge (60 mg of nonsilanol polymeric sorbent functionalized with a quaternary amine sorbent; Waters Corporation, Milford, MA). The column was hydrated with 1 ml of acetonitrile and washed with 1 ml of ddH_2_O before loading 2 ml of the peptide solution previously adjusted to a pH of 10 with 1 mM NaOH. After washing the column with 1 ml of 5% NH_4_OH, we eluted the peptide with 1 ml of assembly buffer and collected the UV-vis spectrum of the column eluant. Peptides eluted from these columns required the addition of a hydrophobe (octadecane, 1:100 aqueous solution) to promote fiber formation.

The standard protocol for evaporation-induced self-assembly started with a 1-ml aliquot of the peptide solution (∼3 mg) containing the hydrophobe (e.g., 1:100 octadecane or C18-silica particles) in assembly buffer and a first round of evaporation for 30 min at 45°C in a Savant SpeedVac concentrator (SPD121P model; Thermo Fisher). Fiber elongation was controlled through refeeding steps every 30 min (four times with 500 μl of the peptide solution or a total of 6 mg of peptide) and reaction mixing by aspiration with a micropipette. At the end of the evaporation process, the dried sample was resuspended in 200 μl of ddH_2_O and dispensed in 50-μl aliquots. The addition of 200 μl ice-cold acetone to each aliquot and overnight incubation at –20°C precipitated the fibers and allowed for their recovery by centrifugation (1 h, 4°C in a microcentrifuge). After a final drying step under a stream of N_2_, the sample was stored at –20°C until further use. When indicated, the final drying step was via lyophilization in order to measure the average particle size of the sample via dynamic light scattering (DLS) in a Malvern Zetasizer Nano-ZS (0.3-nm to 10-μm sensitivity). Assembly efficiency was also calculated as the difference of free PilA_19_ monomer in solution before and after assembly, based on protein concentrations measured by absorbance at 280 nm in a NanoDrop spectrophotometer (Thermo Scientific) using a standard curve of bovine serum albumin (BSA).

### Scanning probe microscopy.

Conductivity studies were conducted in a clean room using PilA_19_ fibers stored dry at –20°C and rehydrated in 200 μl of ddH_2_O at 4°C for 12 to 18 h. Deposition for 10 min of 10-μl aliquots of the solution onto the surface of a freshly cleaved highly oriented pyrolytic graphite (HOPG) (SPI Supplies) promoted the adsorption of the fibers to the electrode. Absorbent paper wicked off excess fluid while two washes with 10 μl of ddH_2_O removed impurities from the HOPG surface. Samples were allowed to dry in a sealed container at room temperature for approximately 10 min before imaging the samples in tapping mode by atomic force microscopy (AFM) with an Asylum Research Cypher S system equipped with an AC240TS tip (Asylum Research). Electrode surface scans of 10 × 10 μm^2^ were used to locate the area of sample deposition and image several fields randomly that best represented the distribution of fibers on the surface. The AFM images were analyzed with the free hand tool of ImageJ to measure the length of the fibers. Conductive probe AFM (CP-AFM) analyses, which measures the transversal conductivity of the sample from the electrons flowing between the conductive AFM tip and the HOPG electrode, followed protocols previously used to measure the conductivity of the native pili (8). For controls, we also conducted AFM topographic and CP-AFM conductivity analyses of the native PilA pili, which were purified as reported elsewhere (8). The ±100 mV ohmic region in the current-voltage (*I*-*V*) plots was fitted to a linear regression line using the Igor Pro 6 software to calculate the electrical resistance of the fibers at biological voltages. Analyses of the asymmetry of the *I-V* plots was via rectification scores (ratio of current recorded at the positive voltage over the negative voltage) calculated at ±100 and ±600 mV, as reported elsewhere (12). A rectification score below 1 indicates asymmetric current flow that favors the electrode-to-tip direction, thus electrons flowing from the fiber toward the external electron acceptor.

Scanning tunneling microscopy (STM) analyses (imaging and spectroscopy) used the same AFM instrument but equipped with a mechanically cut Pt-Ir STM tip (Asylum Research) and operated in STM mode. The quality of the STM tip was tested in scans on the freshly cleaved HOPG surface prior to depositing and scanning the pilus samples (sample voltage of 500 mV; current set point of 350 pA). Sample deposition was with hydrated or glutaraldehyde-fixed PilA_19_ fibers, using previously published protocols for the deposition of hydrated or chemically fixed native PilA pili (28). *I-V* plots collected the current tunneling through individual fibers at a set point of 10 pA, with the tip held at ground, while sweeping the bias voltage (±0.6 V) of the HOPG substrate. Regression analyses of data points in the ±100 mV ohmic region used the Igor Pro 6 software. The asymmetry of the STM *I-V* plots and the material’s electron band gap were assessed in plots of the derivative of the current and voltage data points (*dI/dV*) versus the sample voltage (*V*).

## References

[1] Reguera G, McCarthy KD, Mehta T, Nicoll JS, Tuominen MT, Lovley DR 2005 Extracellular electron transfer via microbial nanowires. Nature 435:1098–1101. doi:10.1038/nature03661. 15973408

[2] Cologgi DL, Lampa-Pastirk S, Speers AM, Kelly SD, Reguera G 2011 Extracellular reduction of uranium via *Geobacter* conductive pili as a protective cellular mechanism. Proc Natl Acad Sci U S A 108:15248–15252. doi:10.1073/pnas.1108616108. 21896750 PMC3174638

[3] Reardon PN, Mueller KT 2013 Structure of the type IVa major pilin from the electrically conductive bacterial nanowires of *Geobacter sulfurreducens*. J Biol Chem 288:29260–29266. doi:10.1074/jbc.M113.498527. 23965997 PMC3795228

[4] Feliciano GT, da Silva AJR, Reguera G, Artacho E 2012 The molecular and electronic structure of the peptide subunit of *Geobacter sulfurreducens* conductive pili from first principles. J Phys Chem A 116:8023–8030. doi:10.1021/jp302232p. 22779741

[5] Feliciano GT, Steidl RJ, Reguera G 2015 Structural and functional insights into the conductive pili of *Geobacter sulfurreducens* revealed in molecular dynamics simulations. Phys Chem Chem Phys 17:22217–22226. doi:10.1039/c5cp03432a. 26243427

[6] Reguera G 2018 Harnessing the power of microbial nanowires. Microb Biotechnol 11:979–994. doi:10.1111/1751-7915.13280. 29806247 PMC6201914

[7] Richter LV, Sandler SJ, Weis RM 2012 Two isoforms of *Geobacter sulfurreducens* PilA have distinct roles in pilus biogenesis, cytochrome localization, extracellular electron transfer, and biofilm formation. J Bacteriol 194:2551–2563. doi:10.1128/JB.06366-11. 22408162 PMC3347174

[8] Lampa-Pastirk S, Veazey JP, Walsh KA, Feliciano GT, Steidl RJ, Tessmer SH, Reguera G 2016 Thermally activated charge transport in microbial protein nanowires. Sci Rep 6:23517. doi:10.1038/srep23517. 27009596 PMC4806346

[9] Speers AM, Schindler BD, Hwang J, Genc A, Reguera G 2016 Genetic identification of a PilT motor in *Geobacter sulfurreducens* reveals a role for pilus retraction in extracellular electron transfer. Front Microbiol 7:1578. doi:10.3389/fmicb.2016.01578. 27799920 PMC5065972

[10] Steidl R, Lampa-Pastirk S, Reguera G 2016 Mechanistic stratification in electroactive biofilms of *Geobacter sulfurreducens* mediated by pilus nanowires. Nat Commun 7:12217. doi:10.1038/ncomms12217. 27481214 PMC4974642

[11] Craig L, Li J 2008 Type IV pili: paradoxes in form and function. Curr Opin Struct Biol 18:267–277. doi:10.1016/j.sbi.2007.12.009. 18249533 PMC2442734

[12] Cosert KM, Steidl RJ, Castro-Forero A, Worden RM, Reguera G 2017 Electronic characterization of *Geobacter sulfurreducens* pilins in self-assembled monolayers unmasks tunnelling and hopping conduction pathways. Phys Chem Chem Phys 19:11163–11172. doi:10.1039/c7cp00885f. 28402361

[13] Cosert KM, Reguera G 2019 Voltammetric study of conductive planar assemblies of *Geobacter* nanowire pilins unmasks their ability to bind and mineralize divalent cobalt. J Ind Microbiol Biotechnol 46:1239. doi:10.1007/s10295-019-02167-5. 30953253

[14] Conchillo-Sole O, de Groot NS, Aviles FX, Vendrell J, Daura X, Ventura S 2007 AGGRESCAN: a server for the prediction and evaluation of “hot spots” of aggregation in polypeptides. BMC Bioinformatics 8:65. doi:10.1186/1471-2105-8-65. 17324296 PMC1828741

[15] Kyte J, Doolittle RF 1982 A simple method for displaying the hydropathic character of a protein. J Mol Biol 157:105–132. doi:10.1016/0022-2836(82)90515-0. 7108955

[16] Xu MQ, Paulus H, Chong S 2000 Fusions to self-splicing inteins for protein purification. Methods Enzymol 326:376–418. doi:10.1016/s0076-6879(00)26066-7. 11036654

[17] Venyaminov S, Baikalov IA, Shen ZM, Wu CS, Yang JT 1993 Circular dichroic analysis of denatured proteins: inclusion of denatured proteins in the reference set. Anal Biochem 214:17–24. doi:10.1006/abio.1993.1450. 8250221

[18] Holzwarth G, Doty P 1965 The ultraviolet circular dichroism of polypeptides. J Am Chem Soc 87:218–228. doi:10.1021/ja01080a015. 14228459

[19] Hjelmeland LM, Chrambach A 1984 Solubilization of functional membrane proteins. Methods Enzymol 104:305–318. doi:10.1016/s0076-6879(84)04097-0. 6232441

[20] Le Maire M, Champeil P, Møller JV 2000 Interaction of membrane proteins and lipids with solubilizing detergents. Biochim Biophys Acta 1508:86–111. doi:10.1016/S0304-4157(00)00010-1. 11090820

[21] Montserret R, McLeish MJ, Bockmann A, Geourjon C, Penin F 2000 Involvement of electrostatic interactions in the mechanism of peptide folding induced by sodium dodecyl sulfate binding. Biochemistry 39:8362–8373. doi:10.1021/bi000208x. 10913242

[22] Cooper TM, Woody RW 1990 The effect of conformation on the CD of interacting helices: a theoretical study of tropomyosin. Biopolymers 30:657–676. doi:10.1002/bip.360300703. 2275971

[23] Albert JS, Hamilton AD 1995 Stabilization of helical domains in short peptides using hydrophobic interactions. Biochemistry 34:984–990. doi:10.1021/bi00003a033. 7827056

[24] Audette GF, van Schaik EJ, Hazes B, Irvin RT 2004 DNA-binding protein nanotubes: learning from Nature’s nanotech examples. Nano Lett 4:1897–1902. doi:10.1021/nl048942f.

[25] Petrov A, Audette GF 2012 Peptide and protein-based nanotubes for nanobiotechnology. Wiley Interdiscip Rev Nanomed Nanobiotechnol 4:575–585. doi:10.1002/wnan.1180. 22753264

[26] Lombardo S, Jasbi SZ, Jeung S-K, Morin S, Audette GF 2009 Initial studies of protein nanotube oligomerization from a modified gold surface. J Bionanosci 3:61–65. doi:10.1166/jbns.2009.1006.

[27] Li J, Egelman EH, Craig L 2012 Structure of the *Vibrio cholerae* type IVb pilus and stability comparison with the *Neisseria gonorrhoeae* type IVa pilus. J Mol Biol 418:47–64. doi:10.1016/j.jmb.2012.02.017. 22361030 PMC3389824

[28] Veazey JP, Reguera G, Tessmer SH 2011 Electronic properties of conductive pili of the metal-reducing bacterium *Geobacter sulfurreducens* probed by scanning tunneling microscopy. Phys Rev E Stat Nonlin Soft Matter Phys 84:060901. doi:10.1103/PhysRevE.84.060901. 22304032

[29] Reguera G 2018 Microbial nanowires and electroactive biofilms. FEMS Microbiol Ecol 94:fiy086. doi:10.1093/femsec/fiy086. 29931163

[30] Ferrone F 1999 Analysis of protein aggregation kinetics. Methods Enzymol 309:256–274. doi:10.1016/s0076-6879(99)09019-9. 10507029

[31] Xue WF, Homans SW, Radford SE 2008 Systematic analysis of nucleation-dependent polymerization reveals new insights into the mechanism of amyloid self-assembly. Proc Natl Acad Sci U S A 105:8926–8931. doi:10.1073/pnas.0711664105. 18579777 PMC2440360

[32] Adler-Abramovich L, Marco P, Arnon ZA, Creasey RC, Michaels TC, Levin A, Scurr DJ, Roberts CJ, Knowles TP, Tendler SJ, Gazit E 2016 Controlling the physical dimensions of peptide nanotubes by supramolecular polymer coassembly. ACS Nano 10:7436–7442. doi:10.1021/acsnano.6b01587. 27351519

[33] Abdolvahabi A, Shi Y, Rasouli S, Croom CM, Chuprin A, Shaw BF 2017 How do gyrating beads accelerate amyloid fibrillization? Biophys J 112:250–264. doi:10.1016/j.bpj.2016.12.004. 28122213 PMC5266089

[34] Yilbas BS, Ali H, Al-Sharafi A, Al-Aqeeli N 2018 Droplet dynamics on a hydrophobic surface coated with N-octadecane phase change material. Colloid Surface A 546:28–39. doi:10.1016/j.colsurfa.2018.02.073.

[35] Goktas NI, Wilson P, Ghukasyan A, Wagner D, McNamee S, LaPierre RR 2018 Nanowires for energy: a review. Appl Phys Rev 5:041305. doi:10.1063/1.5054842.

[36] Tansel B 2017 From electronic consumer products to e-wastes: global outlook, waste quantities, recycling challenges. Environ Int 98:35–45. doi:10.1016/j.envint.2016.10.002. 27726897

[37] Sun H, Zhu J, Baumann D, Peng L, Xu Y, Shakir I, Huang Y, Duan X 2019 Hierarchical 3D electrodes for electrochemical energy storage. Nat Rev Mater 4:45–60. doi:10.1038/s41578-018-0069-9.

[38] Canal Marques A, Cabrera J-M, de Fraga Malfatti C 2013 Printed circuit boards: a review on the perspective of sustainability. J Environ Manage 131:298–306. doi:10.1016/j.jenvman.2013.10.003. 24189538

[39] Abdelbasir SM, Hassan SSM, Kamel AH, El-Nasr RS 2018 Status of electronic waste recycling techniques: a review. Environ Sci Pollut Res Int 25:16533–16547. doi:10.1007/s11356-018-2136-6. 29737485

[40] Coppi MV, Leang C, Sandler SJ, Lovley DR 2001 Development of a genetic system for *Geobacter sulfurreducens*. Appl Environ Microbiol 67:3180–3187. doi:10.1128/AEM.67.7.3180-3187.2001. 11425739 PMC92998

[41] Greenfield NJ 2006 Using circular dichroism spectra to estimate protein secondary structure. Nat Protoc 1:2876–2890. doi:10.1038/nprot.2006.202. 17406547 PMC2728378

[42] Mihalyi E 1968 Numerical values of the absorbances of the aromatic amino acids in acid, neutral, and alkaline solutions. J Chem Eng Data 13:179–182. doi:10.1021/je60037a011.

[43] Wallace BA, Janes RW 2009 Modern techniques for circular dichroism and synchrotron radiation circular dichroism spectroscopy, vol 1. IOS Press, Amsterdam, The Netherlands.

[44] Whitmore L, Wallace BA 2004 DICHROWEB, an online server for protein secondary structure analyses from circular dichroism spectroscopic data. Nucleic Acids Res 32:W668–W673. doi:10.1093/nar/gkh371. 15215473 PMC441509

[45] Whitmore L, Wallace BA 2008 Protein secondary structure analyses from circular dichroism spectroscopy: methods and reference databases. Biopolymers 89:392–400. doi:10.1002/bip.20853. 17896349

[46] Provencher SW 1982 CONTIN: a general-purpose constrained regularization program for inverting noisy linear algebraic and integral-equations. Comput Phys Commun 27:229–242. doi:10.1016/0010-4655(82)90174-6.

[47] van Stokkum IHM, Spoelder HJW, Bloemendal M, van Grondelle R, Groen F 1990 Estimation of protein secondary structure and error analysis from circular dichroism spectra. Anal Biochem 191:110–118. doi:10.1016/0003-2697(90)90396-Q. 2077933

[48] Sreerama N, Woody RW 2000 Estimation of protein secondary structure from circular dichroism spectra: comparison of CONTIN, SELCON, and CDSSTR methods with an expanded reference set. Anal Biochem 287:252–260. doi:10.1006/abio.2000.4880. 11112271

