# Supplementary figures and images for: Bottom-Up Fabrication of Protein Nanowires via Controlled Self-Assembly of Recombinant Geobacter Pilins
Source: mBio. 2019 Dec 10;10(6):e02721-19. doi: 10.1128/mBio.02721-19 (PMC6904877; doi:10.1128/mBio.02721-19)

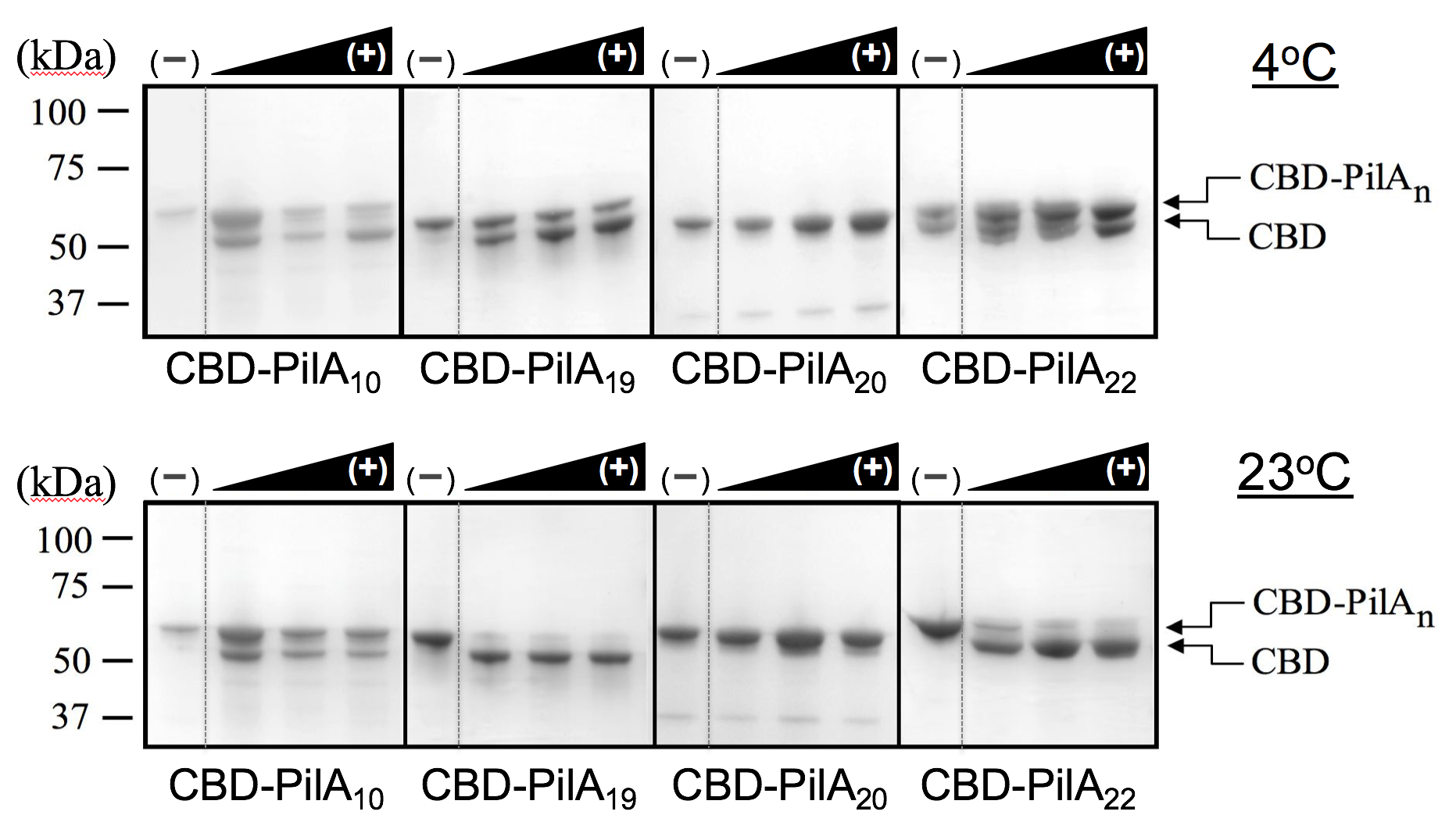

Supplement: FIG S1 [file mBio.02721-19-sf001.tif]

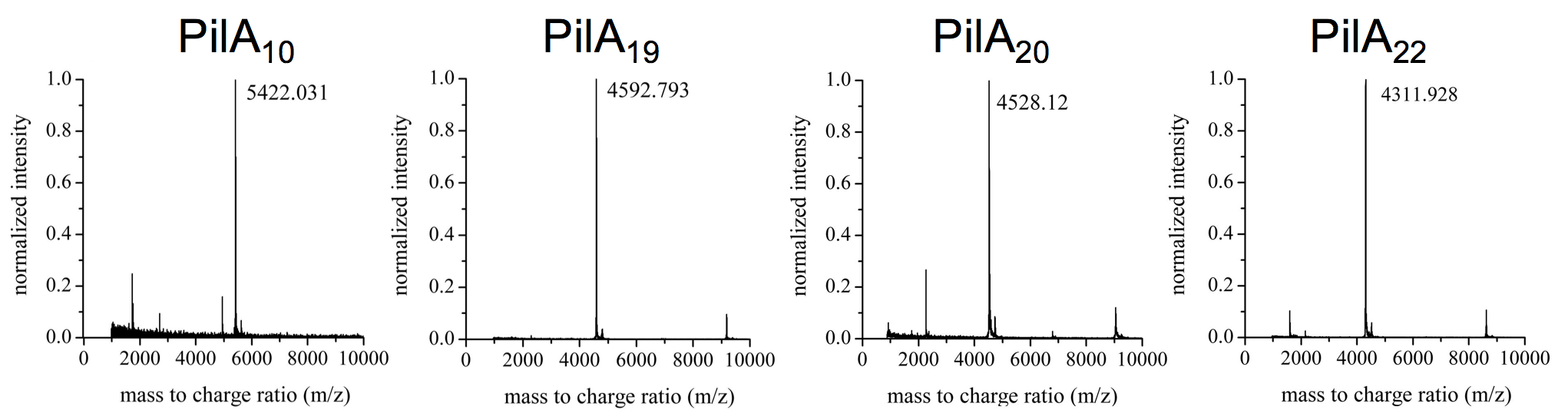

Supplement: FIG S2 [file mBio.02721-19-sf002.tif]

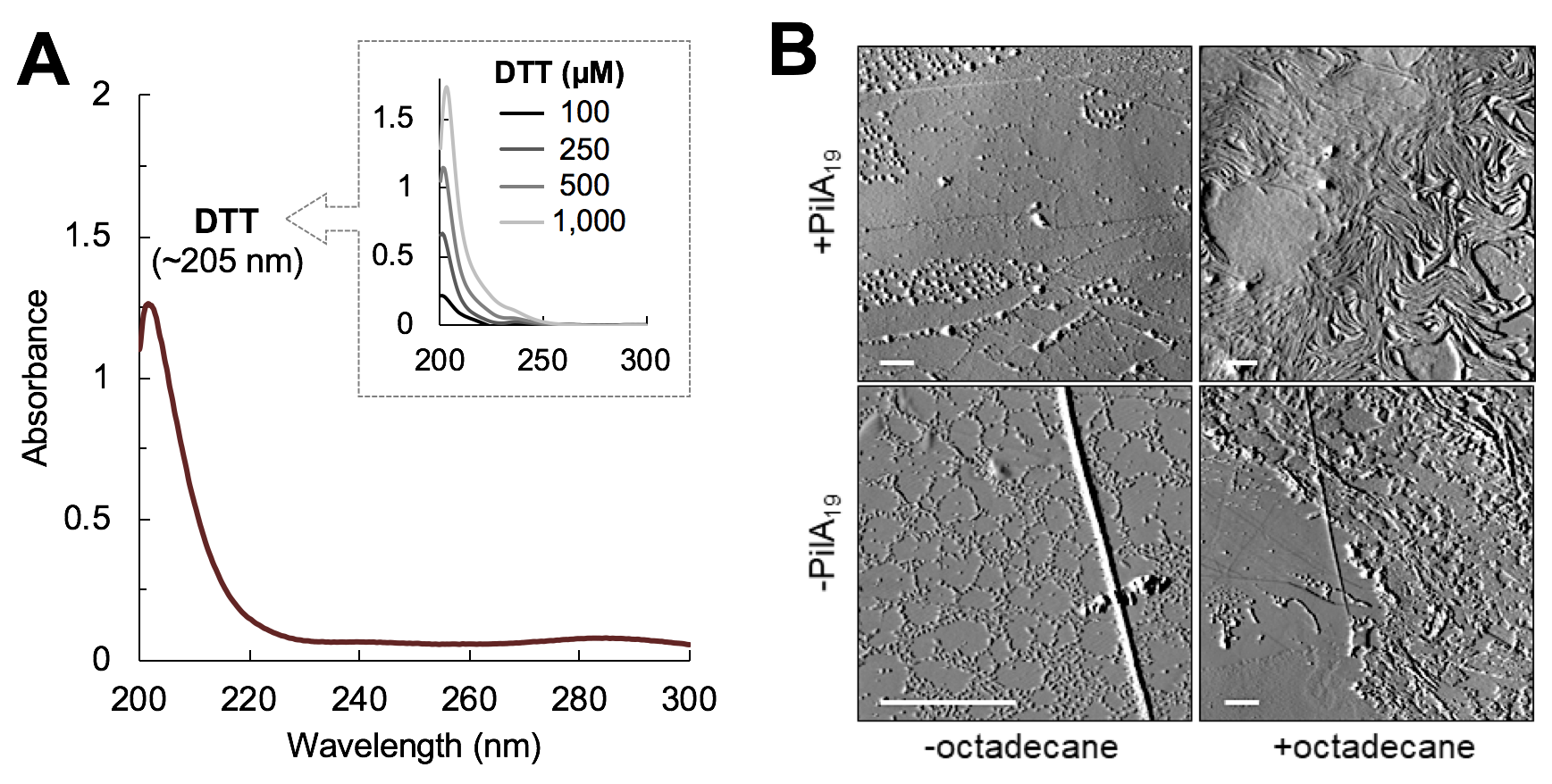

Supplement: FIG S3 [file mBio.02721-19-sf003.tif]

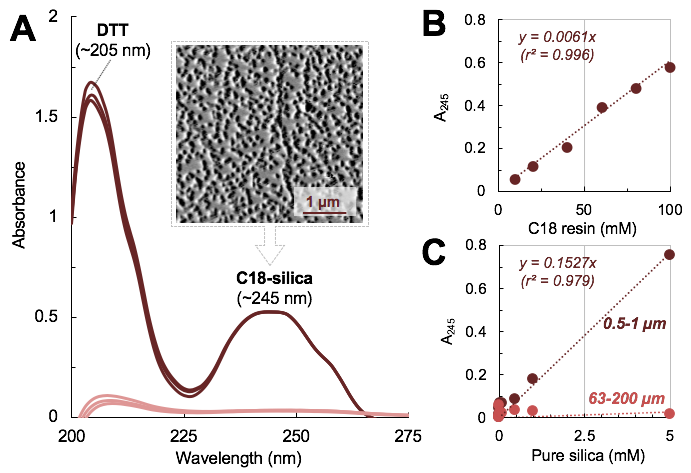

Supplement: FIG S4 [file mBio.02721-19-sf004.tif]

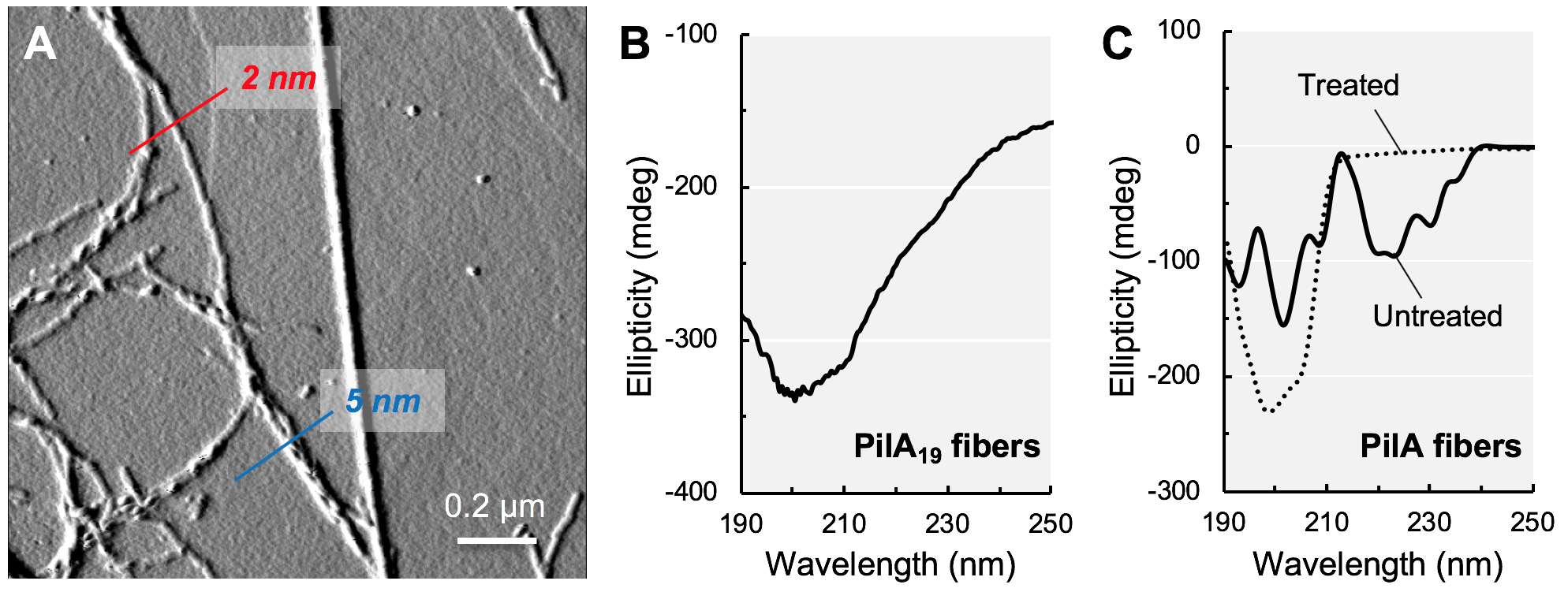

Supplement: FIG S5 [file mBio.02721-19-sf005.tif]
