# Supplementary material for: Bottom-Up Fabrication of Protein Nanowires via Controlled Self-Assembly of Recombinant Geobacter Pilins
Source: mBio. 2019 Dec 10;10(6):e02721-19. doi: 10.1128/mBio.02721-19 (PMC6904877; doi:10.1128/mBio.02721-19)
Supplement: TABLE S1 [file mBio.02721-19-st001.docx]

**Table S1:** Primers (forward and reverse) used to clone the mature *pilA* sequence in the expression vector pTYB11 and PCR-amplified truncated derivatives (*pilA_n_*).

| **Plasmid** | **Primer sequence (5’-3’)***^1^* | **Reference** |
| --- | --- | --- |
| pTYB11*::pilA* | GGTGGTTGCTCTTCCAACTTCACCCTTATCGAGCTGCT | (12) |
|  | GGTGGTCTGCAGTCATTAACTTTCGGGCGGATAGGT |  |
| pTYB11*::pilA10* | GGTGGTCTGCAGTCATTAACTTTCGGGCGGATAGGT | This study |
|  | GGTGGTTGCTCTTCCAACGCGATCATCGGTATTCTCGC |  |
| pTYB11*::pilA19* | GGTGGTTGCTCTTCCAACGCGATTCCGCAGTTCTCGGC | (12) |
|  | GGTGGTCTGCAGTCATTAACTTTCGGGCGGATAGGT |  |
| pTYB11*::pilA20* | GGTGGTTGCTCTTCCAACATTCCGCAGTTCTCGGCGTA | This study |
|  | GGTGGTCTGCAGTCATTAACTTTCGGGCGGATAGGT |  |
| pTYB11*::pilA22* | GGTGGTTGCTCTTCCAACCAGTTCTCGGCGTATCGTGT | This study |
|  | GGTGGTCTGCAGTCATTAACTTTCGGGCGGATAGGT |  |

*^1^*Restriction sites (SapI, GCTCTTC; PstI, CTGCAG) are underlined.
